# Mast Cell: A Multi-Functional Master Cell

**DOI:** 10.3389/fimmu.2015.00620

**Published:** 2016-01-06

**Authors:** Melissa Krystel-Whittemore, Kottarappat N. Dileepan, John G. Wood

**Affiliations:** ^1^Department of Molecular and Integrative Physiology, University of Kansas Medical Center, Kansas City, KS, USA; ^2^Department of Medicine, Division of Allergy, Clinical Immunology and Rheumatology, University of Kansas Medical Center, Kansas City, KS, USA; ^3^Department of Surgery, University of Kansas Medical Center, Kansas City, KS, USA

**Keywords:** mast cell, immune system, inflammatory mediators, systemic hypoxia, atherogenesis

## Abstract

Mast cells are immune cells of the myeloid lineage and are present in connective tissues throughout the body. The activation and degranulation of mast cells significantly modulates many aspects of physiological and pathological conditions in various settings. With respect to normal physiological functions, mast cells are known to regulate vasodilation, vascular homeostasis, innate and adaptive immune responses, angiogenesis, and venom detoxification. On the other hand, mast cells have also been implicated in the pathophysiology of many diseases, including allergy, asthma, anaphylaxis, gastrointestinal disorders, many types of malignancies, and cardiovascular diseases. This review summarizes the current understanding of the role of mast cells in many pathophysiological conditions.

## Introduction

Mast cells are important cells of the immune system and are of the hematopoietic lineage. Mast cells are originated from pluripotent progenitor cells of the bone marrow, and mature under the influence of the c-kit ligand and stem cell factor in the presence of other distinct growth factors provided by the microenvironment of the tissue where they are destined to reside. Under normal conditions, mature mast cells do not circulate in the bloodstream. However, mast cell progenitors migrate into tissues and differentiate into mast cells under the influence of stem cell factor and various cytokines. Mast cells are present throughout the body and they play important roles in the maintenance of many physiological functions as well as in the pathophysiology of diseases. Accordingly, this review is focused on the role of mast cells in a wide range of physiological functions and pathogenesis of a variety of disease states.

### Location of Mast Cells

Mast cells are found in mucosal and epithelial tissues throughout the body. In rodents, mast cells also reside in peritoneal and thoracic cavities. Mast cells are found in all vascularized tissues except for the central nervous system and the retina (1). Mast cells are located at the junction point of the host and external environment at places of entry of antigen (gastrointestinal tract, skin, respiratory epithelium) (1–4). Mast cells are located in areas below the epithelium in connective tissue surrounding blood cells, smooth muscle, mucous, and hair follicles.

The cytoplasm of the mast cell contains 50–200 large granules that store inflammatory mediators, including histamine, heparin, a variety of cytokines, chondroitin sulfate, and neutral proteases (1). In order for mast cells to migrate to their target locations, the co-ordinated effects of integrins, adhesion molecules, chemokines, cytokines, and growth factors are necessary (5). Mast cell progenitors are found in high numbers in the small intestine. CXCR2 expressed on mast cell progenitors directs their migration to the small intestine. Binding of α4β7 integrins (expressed on mast cells) to adhesion molecule VCAM-1 on the endothelium initiates the transit of mast cell precursors out of the circulation (5).

The lungs do not have many mast cell progenitors in a normal physiological state. Upon antigen-induced inflammation of the respiratory endothelium, mast cell progenitors are recruited by engaging α4β7 integrins, VCAM-1, and CXCR2. Additionally, CCR-2 and CCL-2 are involved in the recruitment of mast cell progenitors to the respiratory endothelium. When mature mast cells are activated and degranulated, more mast cell progenitors are recruited to the site of inflammation (5).

There are two phenotypes of human mast cells: mucosal mast cells that produce only tryptase and connective tissue mast cells that produce chymase, tryptase, and carboxypeptidases (6, 7). Mast cell activation and mediator release have different effects in various tissues and organs. Most common sites in the body exposed to antigens are the mucosa of the respiratory tract (airborne), gastrointestinal tract (food borne), blood (wounds, absorption from respiratory tract/gastrointestinal tract), and connective tissues (8).

When the gastrointestinal tract is exposed to an antigen, its response is to increase fluid secretion, increase smooth muscle contraction, and increase peristalsis. Proteins derived from different plants and animals can act as antigens and activate the immune system in vulnerable subjects (8). The antigen (peptide) permeates through the epithelial layer of the mucosa of the gut and binds to IgE on mucosal mast cells. These peptides are presented to Th2 cells, and if there is an IgE antibody against the peptide present, it will cause activation of the mast cell resulting in an immune response. This causes mast cells to degranulate and release a variety of inflammatory mediators. These mediators increase vascular permeability, causing edema in the gut epithelium and smooth muscle contraction, which lead to vomiting and diarrhea. This type of reaction can occur in response to peptides found in certain medications. Food allergens can also cause skin reactions. Uptake from the gastrointestinal tract can introduce antigens into the blood, which are transported throughout the body where they bind to IgE on mast cells in the connective tissue in the deep layers of the skin. This results in urticarial reaction and angioedema (8).

In the respiratory tract, the immune response to mast cell activation results in airway constriction, increased mucous production, and cough (1). The most common introduction of antigens to the respiratory tract is via inhalation. Mucosal mast cells in the nasal epithelium are activated by antigens that diffuse across the mucosa after being inhaled. In the respiratory tract, mast cell degranulation increases vascular permeability and local edema, which can obstruct nasal airways and lead to congestion (9, 10). There is increased production of mucus and its accumulation can block off the sinuses and result in a bacterial infection. Mast cells also play a pivotal role in the pathophysiology of allergic asthma. This is caused by an inflammatory response in the airways, which results from inhaled antigens that get into the lower respiratory tract and cause mast cell degranulation and local inflammation. These events lead to increased vascular permeability, fluid accumulation, and edema, which can obstruct the airways. Bronchial constriction can occur due to smooth muscle contraction, which can lead to airway obstruction that is seen in asthma. Air is, therefore, trapped and total lung capacity is increased while forced expiratory volume in 1 s (FEV1) and forced vital capacity (FVC) are decreased (8). In the blood vessels, increased vascular permeability leads to edema and local inflammation, which results in antigen transport to the lymph nodes (11).

In the skin, antigens, via IgE, activate mast cells in the deep layers of connective tissue. Mast cells release histamine as well as other vasoactive molecules, which cause urticaria (hives). If the antigen activates mast cells in deeper tissue, this can lead to angioedema. If the response is prolonged, atopic dermatitis or eczema may occur. Eczema is seen clinically as a chronic itching skin rash with raised lesions and fluid discharge. Eczema is more commonly seen in childhood while allergic rhinitis and asthma are seen throughout life (8).

### Mechanism of Activation

Mast cells are known for their main mechanism of action: IgE-mediated allergic reactions through the FcϵRI receptor. IgE antibodies are produced by mature B cells in response to CD4+ Th2 cells. Naïve mature B cells produce IgM and IgD antibodies. Once they become activated by an antigen, B cells will proliferate. If these B cells interact with cytokines, such as IL-4 (which is modulated by CD4+ Th2 cells), the antibody class switches from IgM to IgE (12). IgE is mostly found bound to FcϵRI receptors on the mast cell, and very little IgE is found as a soluble antibody in circulation. When an antigen comes in contact with the mast cell, it crosslinks two or more FcϵRI molecules and activates the release of granules from the mast cell (13). IgE is found in the connective tissue under epithelial layers of the skin, in the respiratory tract, and also in the gastrointestinal tract (1). In addition to FcϵRI, mast cells also express Fc receptors for IgA and IgG, receptors for adenosine, C3a, chemokines, cytokines, and pathogen-associated molecular patterns (PAMPs), as well as toll-like receptors (TLRs), all of which are involved in mast cell activation and immune response.

The most common physiological pathway for mast cell activation is via antigen/IgE/FcϵRI cross linking (14). FcϵRI consists of an α-chain that binds to IgE, a β-chain, which spans the membrane, and γ chains, which are a disulfide-linked homodimer. FcϵRI interacts with LYN tyrosine kinase, which phosphorylates the tyrosine in its immunoreceptor tyrosine bases activation motifs (ITAMs) on the B and γ chains of the FcϵRI (15). Lyn activates Syk tyrosine kinases, which phosphorylates signaling proteins, such as LAT1 and LAT2 (linkers for activation of T cells) (16). Phosphorylated PLCγ hydrolyzes phosphatidylinositol-4,5-bisphosphate to make inositol-1,4,5-triphosphate (IP3) and diacylglycerol (DAG). IP3 and DAG are second messengers and IP3 causes calcium mobilization from the endoplasmic reticulum (17). Calcium release activates and causes NFκB to translocate to the nucleus of the cell, which results in transcription of cytokines, such as IL-6, TNFα, and IL-13. Zeb2 is involved in regulation of degranulation upon stimulation via FcϵRI (18). Activation of FcϵRI activates Fyn (Src kinase). Fyn regulates mast cell degranulation, which is complementary to the Lyn signaling pathway. Fyn activates PI3K, which activates Akt and produces PIP3 (15). This activates mTOR, which is involved in mast cell chemotaxis and cytokine production (14). There are also receptors for IgG called FcγR. The y-chain homodimer is the same in FcγRI as in FcϵRI so the signal sent from FcγR can crosstalk with FcϵRI (14). Repeated and controlled exposure of mast cells to antigen can desensitize a patient’s sensitivity. Although the mechanisms are not clearly understood, the slow and persistent degranulation of mast cells is thought to be one of the mechanisms. The desensitization protocol is used in patients who are allergic to certain drugs (e.g., penicillin) but need treatment for a life-threatening bacterial infection that can only be treated with this drug.

Mast cell desensitization can occur from exposure to increasing doses of antigen. This technique can be used if a patient is allergic to a necessary drug and prevention of anaphylactic reactions to food. By desensitizing the receptors, this can decrease the number of FcϵRI molecules available on the mast cell surface (19).

## Physiological Roles of Mast Cells

Mast cells are involved in the regulation of variety of physiological functions, including vasodilation, angiogenesis, bacterial, and parasite elimination. In addition, mast cells regulate functions of many cell types, such as dendritic cells, macrophages, T cells, B cells, fibroblasts, eosinophils, endothelial cells, and epithelial cells. Since, mast cells generate and release multi-potent molecules, such as histamine, proteases, prostanoids, leukotrienes, heparin, and many cytokines, chemokines, and growth factors, they have the capacity to be involved in regulating the functions of many organs and tissues. One of the mostly studied functions of the mast cell is its role in vascular and bronchial homeostasis. Mast cells also play a significant role in the regulation of bone growth, remodeling, and mineral homeostasis.

### Angiogenesis

Mast cells are involved with enhancing angiogenesis (20). Mast cells secrete pro-angiogenic factors, such as VEGF, bFGF, TGF-beta, TNF-alpha, and IL-8. In addition, mast cells release proteases and heparin which release pro-angiogenic factors that bind to heparin. Histamine, released by mast cells, induces permeability of the microvasculature that also induces angiogenesis. There is also evidence of mast cells enhancing angiogenesis in tumor growth (20).

### Homeostasis

Mast cells contribute to homeostasis in the immune system. They serve as a first line of defense against antigens entering the body due to their location in the skin and mucosa (21). Mast cells are especially important in the homeostasis of the commensal bacteria of the gut (22). The digestive system is constantly exposed to different antigens, such as bacteria (commensal and pathologic) and food antigens. The epithelial cells that line the digestive system serve as a barrier to these antigens. Mast cells are important in the differentiation of follicular helper T cells via ATP signaling. As a result, mast cells play a role in IgA maturation and overall homeostasis of the gut bacteria (22).

### Innate and Adaptive Immunity

Mast cells play an important role in innate and adaptive immunity. Mast cells recognize harmful antigens by binding to pathogens directly or associating with PAMPs on the mast cell surface (23). Most commonly the receptors on the mast cells are TLRs and receptors for complement. Once the antigen binds to the receptors on the mast cell, it causes the release of inflammatory mediators, which helps to eliminate the pathogen that activated it. The mechanism for how this happens depends on which PAMP is recognized. TLR2 is activated by Gram-positive bacteria, and to an extent by Gram-negative bacteria and mycobacteria, which cause the mast cell to release cytokines, such as IL-4. TLR4 binds LPS from Gram-negative bacteria, which causes proinflammatory cytokine release (TNFα, IL-1, IL-6) without degranulation (23, 24). On the other hand, the Gram-positive bacterial product peptidoglycan stimulates mast cell degranulation as well as histamine release via TLR2 activation (25, 26). The elimination of bacteria is aided by mast cells by release of inflammatory mediators that increase vascular permeability, increase fluid accumulation, and recruit immune cells, such as eosinophils, NK cells, and neutrophils. Additionally, mast cells directly produce antibacterial products, such as cathelidcidins, defensins, and psidins. Mast cells also contribute to antiviral responses by recruiting CD8+ T cells, which produce IFN-α and IFN-β (27, 28). One of the first identified functions of the mast cell was to produce an anti-parasitic environment when activated by IgE. Release of mediators from the mast cell increases vascular permeability and smooth muscle contraction, which helps to expel the parasites from the gastrointestinal tract by inducing vomiting or diarrhea or from the respiratory tract by coughing (8).

Mast cells are also involved in adaptive immunity. Mast cells process and present antigens via MHCI and MHCII (29). Mast cells activate dendritic cells that also function as antigen-presenting cells. When mast cells are stimulated through TLF-7, they release IL-1 and TNFα, which causes dendritic cells to move from their location in the skin and go to local lymph nodes and activate cytotoxic T cells. Additionally, mast cells release TNFα, which can activate cytotoxic T cells directly (30).

### Activation and Mediator Release

Mast cells upon activation release preformed and newly synthesized mediators in a phasic fashion. A variety of endogenous and exogenous agents can stimulate mast cells to release mediators immediately. Activation of mast cells occurs when an antigen crosslinks IgE molecules that are bound to FcϵRI on the surface of the mast cell. FcϵRI receptor for IgE has an affinity 100 times greater for the Fc of IgE than of IgG. Because of this, IgE is found bound to the FcϵRI receptors on the mast cell even when there are no antigens present. As a result, this makes the response of the mast cell to an antigen very fast. FcϵRI signaling uses the Lyn-dependent phosphorylation of ITAMs on the B and y subunits of the FcϵRI (1). Protein kinase Syk is activated and autophosphorylated after being recruited to the ITAMs. Syk phosphorylates linker activation of T cells (LAT) and non-T cell activation linker (NTAL). LAT phosphorylates PLC, which produces IP3 and DAG, which activates intracellular calcium influx and PKC activation. NTAL activates PI3K, which also helps with calcium release. This results in degranulation of the mast cells, lipid mediator production, and cytokine production (1).

There are two ways to crosslink IgE molecules on the mast cell surface. If an antigen has the same epitopes, they will crosslink IgE molecules of the same specificity. If an antigen has more than two epitopes, they will crosslink IgE molecules with different specificities (8).

Degranulation occurs a few seconds after crosslinking and results in release of the inflammatory mediators that are stored in the granules (31). Many of the mediators that are stored or newly synthesized by the mast cells attract leukocytes (eosinophils, basophils, Th2 lymphocytes, neutrophils) to the inflammatory site and amplify the inflammatory response (1). The inflammatory mediators increase the permeability of the blood vessels so that the immune cells can move from the blood stream to the affected tissue. After degranulation, mast cells resynthesize the mediators and repopulate granules (8).

Mast cells express TLRs 1–7 and 9, NOD-like receptors (NLRs), and retinoic acid-inducible gene 1 (1). If a TLR on the mast cell is activated, MyD88 and MAL/TIRAP are associated and promote NFκB translocation to the nucleus resulting in cytokine transcription (32). TLR4 can be activated by LPS from Gram-negative bacteria. This causes cytokine production without degranulation. When TLR2 is activated by peptidoglycan, this results in degranulation and cytokine production (33).

**Figure 1:**
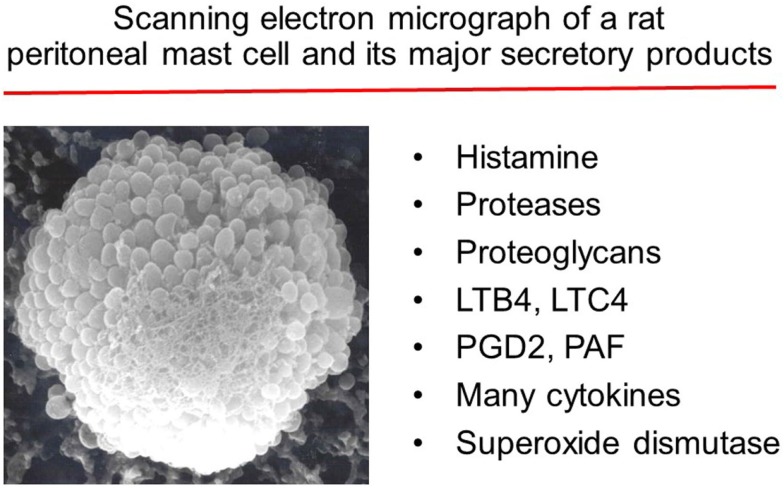
**Major substances released by activated mast cells**.

IgE-mediated activation by FcϵRI causes degranulation and synthesis of many immune mediators, such as eicosanoids and cytokines, as well as other products (Figure 1). When the mast cell is activated, it immediately releases prepackaged granules. Mast cell granules (MCG) can be compared to lysosomes in that there is a low pH and lysosomal enzymes, such as β-hexosaminidase and caspase-3 (34). Tryptase, chymase, cathepsin G, and carboxypeptidase are proteases stored in prepackaged granules that activate metalloproteases in the extracellular matrix. Activation of the metalloproteases breaks down extracellular matrix proteins and remodels the connective tissue matrix. Chymase cleaves fibronectin and collagen by activation of MMPs. β-tryptase has been shown to cleave IgE once the mast cell has been activated to down regulate the allergic response (35). Histamine and heparin are also stored in prepackaged granules and are involved with vascular permeability and smooth muscle contraction. Histamine is the most important mediator released from the mast cell involved with an allergic response. Histamine is derived from the amino acid histidine and works through three different receptors (H1, H2, H3). Stimulation of H1 receptors by the binding of histamine induces the classic allergic reaction. H1 receptors are found on smooth muscle cells and endothelial cells. Activation of H1 receptors on endothelial cells results in increased vascular permeability and activation of smooth muscle cells resulting in contraction, constriction of airways, and mucous secretion (8). TNFα, also stored in the MCG, activates macrophages, endothelium, and cytokines (36). TNF-α binds to endothelial cells and results in increased adhesion molecule expression. Leukocytes can bind to these adhesion molecules and then are brought to the site of inflammation (36, 37).

Other molecules are synthesized and released after the mast cells have been activated. IL-3, IL-5, and GM-CSF are involved with eosinophil production and activation. CCL3 is a chemotactic factor for macrophages and neutrophils (1). Eicosanoids (prostaglandins, leukotrienes, and thromboxanes) are produced by catalytic conversion of arachidonic acid by the action of phospholipase A2 on membrane phospholipids. Mast cells express COX1 and COX2, which converts arachidonic acid into prostaglandins and thromboxanes with the action of specific isomerases (38). Prostaglandins increase vascular permeability and attract neutrophils. Leukotrienes are involved with smooth muscle contraction, airway constriction, and mucous secretion (39). Eicosanoids act at the local area of mast cell degranulation. Platelet-activating factor is released after mast cell activation that acts as a chemotactic factor for leukocytes, and activates neutrophils, eosinophils, and platelets (40). All of the mediators released upon activation results in increased vascular permeability, smooth muscle contraction, and airway constriction. These adaptations can remove parasites from the gastrointestinal tract. Due to the increased vascular permeability, increased fluid in the tissue can enhance elimination of parasites. IgE-mediated mast cell activation would result in physical expulsion of parasites. However, developed countries rarely have parasite infections. Persistent mast cell degranulation resulting from recurrent responses to innocuous substances leads to allergies, asthma, and food allergies. Mast cell degranulation also occurs from grass, pollen, or shellfish-derived allergens. It is of interest that developing countries do not have a high prevalence of allergies or asthma potentially due to childhood exposure to a variety of environmental PAMPs and subsequent desensitization (8).

## Role of Mast Cells in Cardiovascular Disease

Mast cells are important cells of the immune system. The following sections discuss the emerging evidence on the role of mast cells in vascular inflammation during systemic hypoxia and ischemia/reperfusion as well as the progression of atherosclerosis.

### Microvascular Inflammation in Systemic Hypoxia

Mast cells mediate microvascular inflammatory response to systemic hypoxia caused by a reduction in the level of inspired oxygen (41). During hypoxia, leukocytes interact with endothelial adhesion molecules, resulting in leukocyte rolling and adherence within systemic venules, and eventually leukocyte emigration into the tissue. This microvascular inflammatory response was also associated with increased vascular permeability to plasma proteins during systemic hypoxia (42).

Steiner et al. concluded that activation of mast cells contributes to development of a chemotactic gradient during systemic hypoxia and that this is a critical event in the subsequent development of microvascular inflammation (41). This was based on several lines of evidence: 1) systemic hypoxia caused mast cell degranulation, as shown by an increase in ruthenium red uptake in mast cells, 2) the mast cell stabilizer cromolyn attenuated the increases in leukocyte adherence and vascular permeability during systemic hypoxia, and 3) the mast cell activator compound 48/80 caused mast cell degranulation and microvascular inflammation.

The mechanism responsible for mast cell activation during systemic hypoxia is complex, and is partially dependent on increased generation of ROS. Administration of the antioxidant lipoic acid prevents hypoxia-induced mast cell degranulation (37). Mast cell activation also contributes to increased ROS generation during systemic hypoxia, as dihydrorhodamine-dependent fluorescence in venular endothelium was attenuated by cromolyn. Although decreased oxygen levels in culture media have been shown to increase ROS generation in various cells *in vitro* (43), decreased tissue oxygen levels in systemic organs are not the major cause of mast cell degranulation *in vivo*. Dix et al. developed a system to control the tissue oxygen level with the cremaster (the skeletal muscle surrounding the testes) independent of oxygen levels within systemic arterial blood. A local reduction in tissue oxygen levels to that seen during systemic hypoxia did not cause mast cell degranulation or increased leukocyte adherence within cremaster venules (44). During systemic hypoxia, however, these events occurred in the cremaster even though the tissue oxygen levels were maintained at normal levels.

After demonstrating that a proinflammatory mediator was released into the circulation during systemic hypoxia that caused mast cell degranulation and microvascular inflammation, we began a series of studies to find the source of this mediator as well as its identity. Following a reduction in inspired oxygen, the first organ to become hypoxic is the lungs. Chao et al. found that hypoxia causes release of MCP1 from alveolar macrophages in the lungs, and that increased circulating levels of MCP1 result in mast cell degranulation and microvascular inflammation within systemic organs (45).

### Ischemia/Reperfusion

Ischemia/reperfusion injury occurs following a prolonged decreased blood flow to an organ, followed by the restoration of blood flow. Organ transplantation is the classic example of ischemia/reperfusion injury in which the organ receives no blood flow for a period of time until vessels are re-anastomosed in the recipient. Tissue oxygen levels decrease in the organ during the ischemic phase, but microvascular inflammation does not develop until the reperfusion phase (46). During the ischemic phase, vasodilators accumulate within the tissue resulting in higher than normal blood flow for a period of time when blood flow is restored to the organ. During the reperfusion phase, increased generation of ROS occurs resulting in mast cell activation. The mechanism responsible for mast cell degranulation during ischemia/reperfusion differs from that triggered by allergens, which is mediated by the IgE receptor (FceRI) pathway (47).

During reperfusion, complement molecules C3a and C5a cause mast cell degranulation when activating G-protein-coupled receptors (GPCR) on the mast cell surface (48). Additionally, reactive oxygen species are generated when tissue is reperfused, which activates intracellular pathways causing mast cell degranulation (49). Intracellular and extracellular pathways converge and result in phospholipase C-mediated production of IP3 and DAG, causing calcium release from the endoplasmic reticulum and activation of protein kinase C, leading to degranulation of the mast cell (50, 51).

Mast cells release mediators that increase both leukocyte adhesion to the venular endothelium and vascular permeability during reperfusion (47). These effects are mediated by various substances released by mast cells, such as histamine, tryptase, and chymase (46). Yang et al. examined ischemia in rat liver (46). Rat livers were subjected to 1 h ischemia followed by 24 h of reperfusion. Mast cell degranulation was monitored by toluidine blue staining and assessment of mast cell tryptase. Mast cell degranulation was highest at 2 h of reperfusion, while liver damage was greatest after 6 h of reperfusion. Administration of either the mast cell stabilizer cromolyn or the mast cell activator compound 48/80 to decrease the number of MCG prior to ischemia/reperfusion decreased the severity of hepatic injury (46).

Cardiac arrhythmias, such as ventricular tachycardia and ventricular fibrillation, can develop as a result of mast cell degranulation following ischemia/reperfusion. Activated cardiac mast cells release renin, which converts angiotensinogen to angiotensin 1, which is then formed to angiotensin 2 by angiotensin-converting enzyme (ACE). Angiotensin 2 enhances the release of norepinephrine from nerve endings and can lead to arrhythmias (52). Mast cell stabilizers, such as cromolyn, inhibit this process and, therefore, can help to abolish arrhythmias during myocardial ischemia/reperfusion. It is possible to inhibit mast cells by either inhibiting degranulation or blocking the action of the components that are released during mast cell degranulation (53). Histamine antagonists, ACE inhibitors, and angiotensin 2 receptor blockers are all drugs that inhibit components produced by mast cells. Other drugs that block individual components released during mast cell degranulation are still being investigated (47). Possible targets for inhibition include protein kinase C, which is an important enzyme in the degranulation pathway (54).

### Atherosclerosis

Atherosclerosis is an inflammatory process that involves innate and adaptive immunity (55). During atheroma formation, increased numbers of T cells and macrophages are recruited to the vessel wall (56). Innate immunity involved identification of PAMPs, which generate an inflammatory response via the NFκB pathway (57). Microbial products can contribute to plaque growth. For example, LPS in Gram-negative bacteria can act on endothelial cells and activate TLR expressed on plaques (58). Angiotensin II has been shown to induce cytokine expression in atheroma-related cells (59). Leukocytes are targeted to the site of atheroma from chemokines. Mast cells are recruited to the site of atheroma by eotaxin (60). Macrophages and T cells outnumber mast cells in the atheroma. However, mast cells are important in activation of plaques due to their proteases, which are released leading to plaque rupture and acute coronary syndromes (58). Mast cells are also able to degrade the extracellular matrix of cells in the atheroma and modify lipoproteins (61). When a plaque is physically disrupted, a thrombus can form. Rupture of the fibrous cap of the plaque allows tissue factor from within the intima to come in contact with coagulation factors. Mast cell proteases degrade collagen in the fibrous cap of the plaque, which can lead to plaque rupture (58).

Unstable angina and myocardial infarctions are caused by rupture of atherosclerotic plaques (62). Mast cells play a large role in the pathogenesis of plaque rupture and accumulate in the rupture prone region or human atheromas. Activated mast cells release tryptase and chymase, which are proteases that are found at the rupture site. Plaque destabilization in coronary arteries can occur from inflammatory response caused by mast cells. It is known that systemic activation of mast cells occurs during atherogenesis and results in increased progression of plaques in apoE-deficient mice (62). Mast cell stabilization by cromolyn has been shown to prevent pathophysiological events, such as plaque rupture (62). Systemic mast cell activation leads to plaque progression during atherogenesis as seen with treatment with dinitrophenyl-albumin (DNP), which causes activation in antigen-sensitized mast cells. When mast cells were challenged with DNP in mice, there was an increase in hemorrhage in the plaque. These findings are not seen in mast cells pretreated with cromolyn. This is consistent with findings in humans by Laine et al. (63) who showed that in mice treated with DNP, there was increase in apoptosis of intimal cells. Macrophage apoptosis was most often seen in the center of the atheroma. This leads to increase size of the necrotic core and release of apoptotic microbodies, which increase instability of the plaque and increase thrombosis. Protease inhibitors prevented macrophage apoptosis induced by mast cells. Additionally, macrophage apoptosis was completely inhibited by a H1 receptor antagonist. Mice treated with DNP also had increased vascular permeability, capillary leakage, and increased leukocyte adhesion in the atherosclerotic plaques. Mast cell stabilization with cromolyn prevented acute coronary syndromes (62).

Inflammatory cells, such as mast cells, neutrophils, NK cells, monocytes, macrophages and dendritic cells, play a key role in the development and progression of atherosclerosis (64). LDL and LPS activate these inflammatory cells. Inflammatory cells respond to tissue injury, which results in an inflammatory process. Mast cells are found in all vascularized tissues except for the central nervous system and the retina. Mast cells are located in the intima of carotid arteries and in the shoulder region of atherosclerotic plaques. Mice deficient in FceRIa have decreased lipid deposition in the aortic arch in ApoE−/− mice (65). Reduction in lipid deposition was caused by a decrease in FceRIa-mediated mast cell activation and a decrease in inflammatory mediator release. LDL–IgG complexes have been recognized in plaques in animal model and can activate mast cells resulting in the release of IL-8, TNFα, histamine, and tryptase. Mast cells have also been shown to activate TLR4 that leads to smooth muscle cell apoptosis in the plaque resulting in plaque destabilization (66).

ApoE−/− mice show tryptase overexpression resulting in an increase in the area of carotid plaques and increase in carotid artery stenosis (64). Mast cell tryptase plays a role in leukocyte recruitment. Additionally, it increases MCP-1 and IL-8 production that attracts monocytes and neutrophils in ApoE−/− mice (67). Tryptase increases foam cell formation by inhibiting activation of LXR-α and inhibiting reverse cholesterol transport (68). Tryptase can break down fibronectin and collagen type IV, which can result in plaque rupture and thrombosis. Mast cell chymase can also cause smooth muscle cell apoptosis resulting in destabilization of the plaque (64).

Mast cells are also found in the media and adventitia of the aorta and contribute to development of aneurisms. Risk factors for abdominal aortic aneurisms (AAA) are male gender, advanced age, history of smoking, and atherosclerosis. In AAA, there is inflammation in the media and adventitia of the aorta versus atherosclerotic disease, which is mostly found in the intima. During development of AAA, there is an imbalance of the matrix buildup and breakdown, which leads to weakening of the wall of the aorta and dilation of the aorta. There are many important cells involved in the development of AAA, which are neutrophils, smooth muscle cells, and aortic mast cells (69). Mast cells are involved in degradation of the extracellular matrix (by activation of metalloproteases), apoptosis of smooth muscle cells, and activation of the renin angiotensin system. Experimentally induced AAA in animals can be done with intra-aortic elastase infusion, topical treatment of aorta with CaCl_2_, or angiotensin infusion (70). Sun et al. showed that mast cell deficient mice did not develop AAA (71). Furthermore, rats deficient in mast cells had a lower response to CaCl_2_-induced AAA (72). Histamine, a major mediator of mast cells, activates JNK pathways. Phosphorylated JNK is increased in human AAA and this pathway accelerates degradation of the extracellular matrix. Many studies have shown that subcutaneous injection of angiotensin II causes AAA in animals. Patients treated with ACE inhibitors were less likely to be admitted for ruptured AAA (73). ACE inhibitors prevent AAA in rats infused with elastase (74). Tissue repair in AAA depends on smooth muscle cells that make collagen. However, in human AAA, mast cells can cause smooth muscle cell death by apoptosis via TNFα release. Granzyme B, also released by mast cells, can induce smooth muscle cell apoptosis (75). Additionally, chymase released by mast cell inhibits collagen synthesis from smooth muscle cells. Drug targeting of mast cell mediators of AAA could help in treatment by inhibiting the growth of small AAA before they require surgery (70).

Increased numbers of mast cells are seen during the progression of atherosclerosis (76). They are predominately seen in the intima and adventitia. Mast cells are recruited to the plaque via chemokine CCL-11, which is expressed in the plaque, and CCR-2 which is expressed on the mast cell surface. Mast cells in plaque are located near microvessels (77, 78). When mast cells degranulate, they release histamine and matrix degrading proteases, which can cause microvessel leakiness and rupture leading to intraplaque hemorrhage. Mast cell activation during atherosclerosis was shown to increase the size of the plaque in the brachiocephalic artery of apoE-deficient mice (62). This response was prevented by administration of cromolyn. Another study showed that mast cell deficiency inhibited development of atherosclerotic plaque in LDL receptor-deficient mice (79). Mast cells can be seen as effector cells to induce plaque formation and progression. Overexpression of mast cell tryptase in mice had a greater risk of intraplaque hemorrhage (67). Additionally, a patient cohort study found that serum chymase levels were higher in patients with coronary heart disease. Chymase can modify HDL, affect cholesterol efflux ability, and also enhance the production of Angiotensin II, which is a proatherogenic factor. Chymase also induces apoptosis of vascular smooth muscle cells and endothelial cells (80–82). Activation of mast cells promotes enhanced lipid uptake by macrophages. Heparin-bound LDL is phagocytosed by macrophages to form foam cells (83, 84).

Mast cell activation during plaque development leads to progression and increased leukocyte infiltration and lipid accumulation. The resulting leakiness of microvessels in advanced unstable lesions can lead to hemorrhage of the plaque or rupture of the fibrous cap. This can then result in thrombosis and acute cardiovascular events. Mast cell activation in the plaque can be through IgE-dependent or IgE-independent pathways. IgE levels are high in patients with angina pectoris (85). However, another study showed that plasma IgE levels did not correlate with disease progression or mast cell numbers in Western populations (86). This suggests that mast cell activation in the progression of atherosclerotic plaques may be initiated by another mechanism, such as plaque lipids, which can activate mast cells in the vessel wall. Another mechanism of activation is through C5a activation via C5aR on mast cells. Activated complement is found within the plaque. Activation of mast cells with C5a resulted in an increase in vein graft atherosclerosis, which was inhibited by cromolyn (87). Activation of mast cells can be accomplished via neuropeptides, such as substance P, as mast cells are in close proximity to nerve fibers. Use of mast cell stabilizers for halting plaque progression would be a reasonable treatment option (87).

Mast cells are increased in coronary arteries during spasm and in the rupture prone shoulders of coronary atheromas (63). Risk factors, such as oxidized LDL, reactive oxygen species, complement 5a, substance P, endothelin-1, and thrombin can activate mast cells (87–92). Mast cells synthesize and secrete histamine, proteases, prostaglandin D2, leukotrienes, heparin, and a variety of cytokines, many of which are implicated in CVD (36, 93–100). Furthermore, mast cells enhance endothelial inflammatory responses through upregulation of innate immune mechanisms (101, 102). The clinical significance of mast cells in CVD is evident from their increased presence in the adventitia of coronary arteries of patients with atherosclerosis (98, 103–107). An increase in the number of mast cells is also found to be associated with thrombus formation (108). Endothelial cells can endocytose MCG *in vitro* (107, 109) and *in vivo* (98). MCGs are also involved in the induction of human microvascular endothelial cell proliferation (110), LDL uptake by macrophages, and foam cell formation (111, 112). Although these findings suggest an important role for mast cells in CVD, the mechanism by which mast cell products promote atherogenesis and CVD is not well understood. Others and we have shown that mast cell deficiency attenuates progression of atherosclerosis in ApoE−/− (113) or LDLr−/− (79, 114) mice. Our data also show that mast cell deficiency significantly reduces serum cholesterol, LDL, HDL, IL-6, and IL-10, the expression of COX2 in the aortic tissue, the systemic production of PGI_2_, and infiltration of macrophages and lymphocytes into the plaque in ApoE−/− mice (113).

**Figure 2:**
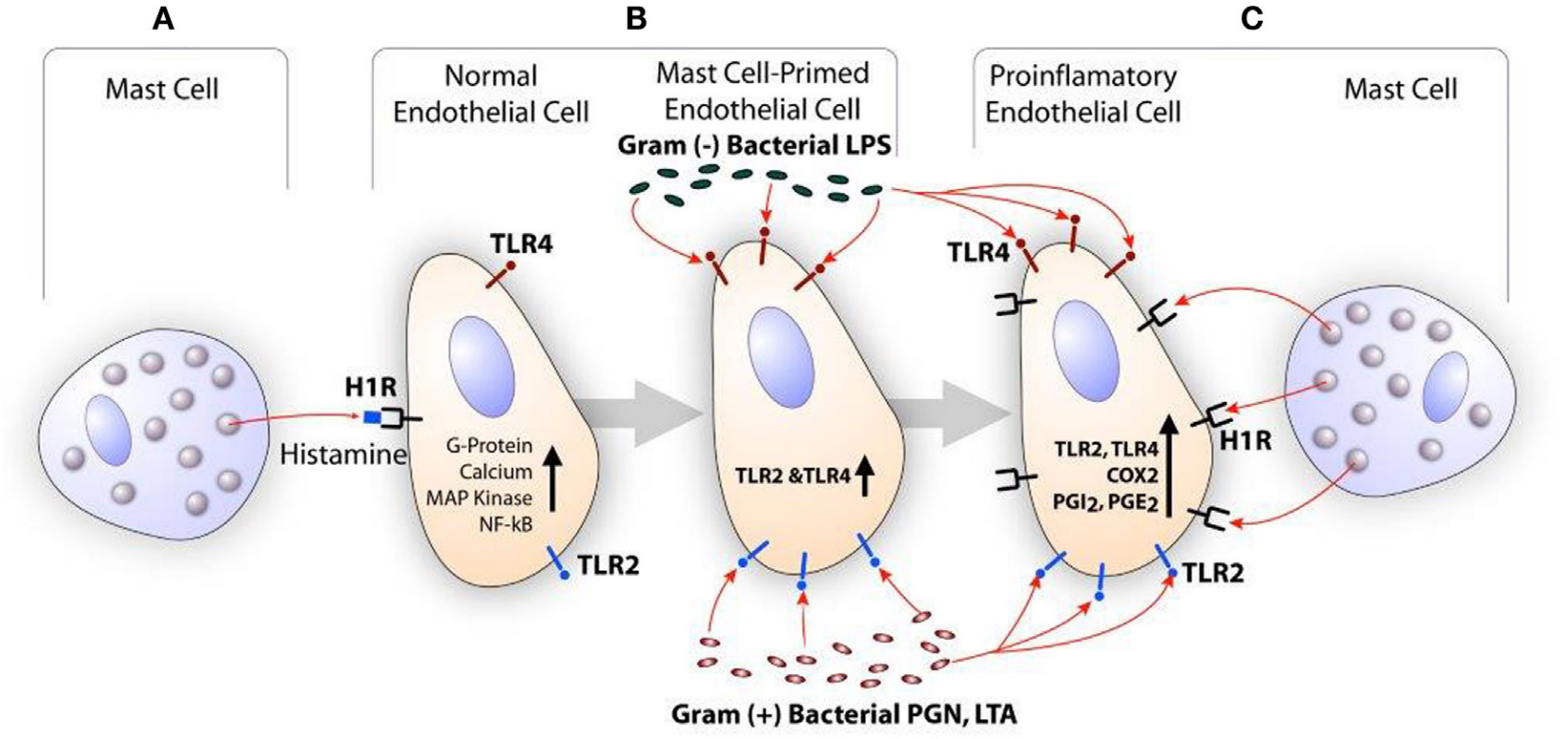
**Scheme showing the synergistic activation of inflammatory response in endothelial cells by mast cell-derived histamine and bacterial products**. **(A)** Histamine secreted by the mast cell stimulates H1R on endothelial cells. **(B)** H1R-mediated endothelial cell activation leads to increased expression of TLR2 and TLR4, and become hyperresponsive to the TLR ligands leading to enhanced inflammatory response. **(C)** Increased TLR2 and TLR4 signaling increases H1R expression. Finally, collective actions of newly expressed TLR2/TLR4 and H1R lead to increased COX2 expression and other proinflammatory changes in the endothelium resulting in persistent vascular inflammation.

Histamine is a major secretory product of the mast cell and is recognized for its role in the regulation of vasodilation and bronchoconstriction (115, 116). Histamine also regulates functions of monocytes and macrophages (117, 118), eosinophils (117, 118), T cells (119), neutrophils, and endothelial cells (120, 121). Depending on the cell types, histamine acts through a family of four distinct GPCR termed H1R, H2R, H3R, and H4R (122). GPCR undergoes desensitization after phosphorylation by GPCR kinase (GRK) after stimulation by the agonist. GRKs are a group of seven mammalian serine and threonine protein kinases (123). GRK2 is one of the members of this group that is known to desensitize H1R and limits its signaling (124, 125). Endothelial cells and smooth muscle cells highly express H1R and this receptor facilitates histamine-mediated inflammatory and hypersensitivity responses (121, 126). The clinical significance of mast cell-derived histamine in CVD is evident from the finding that coronary arteries of patients with ischemic heart disease contain more mast cells and histamine than normal vessels (103), and patients with variant angina have elevated levels of histamine in their coronary circulation (127). Our studies show that histamine acting through H1R stimulates the expression of TLR2, TLR4, IL6, COX2, PGI_2_s, and PGE_2_s genes leading to enhanced production of IL-6, PGE_2_, and PGI_2_ by HCAEC (121, 128). Reports have suggested that histamine induces smooth muscle cell migration and proliferation (129, 130), and regulates intimal thickening model (131). In regard to H1R and atherosclerosis, increased H1R mRNA expression has been reported in smooth muscle cells of intima/media in the atheroma (132). Histamine also increases endothelial cell responses to TLR2 and TLR4 ligands by increasing the expression of these two innate immune receptors (121, 128, 133). We have also shown that LPS induces the expression of functionally active H1R in HCAEC, and enhances sensitivity to histamine (134). These findings suggest that histamine and bacterial agents act in a bidirectional manner amplifying inflammatory responses *via* upregulation of H1R and TLR2/TLR4 (Figure 2).

Histamine induces the production of proinflammatory cytokines, such as IL-6 and IL-8, and anti-atherogenic eicosanoids (PGI2 and PGE2) (121, 128, 133–135). Therefore, it is unclear whether H1R signaling of histamine is proatherogenic or cardioprotective. Some studies show that H1 antihistamines reduce atherogenesis in apoE-deficient mice (136, 137). Raveendran et al. examined apoE−/− mice treated with low or high cetirizine or fexofenadine doses and assessment of atherosclerotic plaques via histological section of the aorta (135). Increased atheroma formation and lesion area were noted in mice with low doses of cetirizine or fexofenadine. This was not associated with increased macrophage, mast cell, or T lymphocyte count. Reduction in the number of mast cells may be due to increased degranulation. However, high doses of cetirizine and fexofenadine did not increase atherosclerosis compared to the control. Ingestion of H1 antihistamines did not alter H1R expression in the plaque area as determined by immunofluorescence. At high doses, the antihistamines may bind to other receptors, such as H4R, which may result in the different response seen than binding H1R. Therefore, antihistamines continue to show a mixed picture with respect to atherosclerosis (135). It should be noted that the vasodilatory effects of histamine may promote lipid accumulation in the vessel wall.

## Conclusion

In summary, mast cells play a key role in regulation of normal physiological processes as well as in many pathophysiological settings. Considerable progress has been made in our understanding of these immune cells in recent years. Additional efforts to define the complex interactions of mast cells will potentially lead to novel clinical approaches for many pathological conditions.

## Conflict of Interest Statement

The authors declare that the research was conducted in the absence of any commercial or financial relationships that could be construed as a potential conflict of interest.
